# Chlorido{*N*,*N*′-*o*-phenyl­ene-[6,6′-ethyl­enebis(pyridine-2-carboxamide)]}iron(III)

**DOI:** 10.1107/S1600536808041354

**Published:** 2008-12-10

**Authors:** Li Yang, Bin Tang

**Affiliations:** a Department of Chemical Engineering, Yibin University, Yibin 644000, People’s Republic of China; b Department of Chemistry, Luzhou Medicial College, Luzhou 646000, People’s Republic of China

## Abstract

In the title compound, [Fe(C_20_H_14_N_4_O_2_)Cl], the Fe^III^ ion is in a distorted square-pyramidal environment, with two pyridine and two deprotonated amide N atoms in the basal plane and the Cl ion in the apical position. The Fe^III^ ion is displaced from the basal plane of the square- pyramid towards the apical Cl atom by 0.2942 (4) Å. The mol­ecules are linked into a three-dimensional network by C—H⋯Cl and C—H⋯O hydrogen bonds.

## Related literature

For general background, see: Liu *et al.* (2006 ▶); Yang *et al.* (2007 ▶); Momenteau & Reed (1994 ▶). For related structures, see: Rath *et al.* (2004 ▶); Xu *et al.* (2007 ▶).
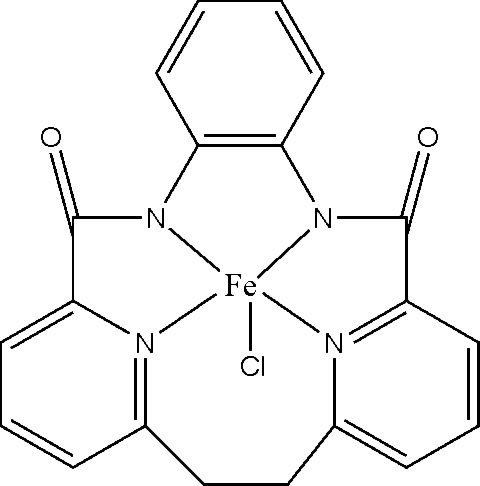


## Experimental

### 

#### Crystal data

[Fe(C_20_H_14_N_4_O_2_)Cl]
*M* *_r_* = 433.65
Monoclinic, 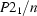

*a* = 11.8532 (2) Å
*b* = 8.2028 (1) Å
*c* = 19.3507 (3) Å
β = 106.889 (1)°
*V* = 1800.31 (5) Å^3^
*Z* = 4
Mo *K*α radiation
μ = 1.01 mm^−1^
*T* = 296 (2) K
0.44 × 0.16 × 0.10 mm

#### Data collection

Bruker SMART CCD area-detector diffractometer
Absorption correction: multi-scan (*SADABS*, Sheldrick, 1996 ▶) *T*_min_ = 0.778, *T*_max_ = 1.000 (expected range = 0.703–0.904)
24687 measured reflections
4142 independent reflections
3415 reflections with *I* > 2σ(*I*)
*R*_int_ = 0.041

#### Refinement

*R*[*F* ^2^ > 2σ(*F* ^2^)] = 0.040
*wR*(*F* ^2^) = 0.126
*S* = 1.01
4142 reflections
253 parameters
H-atom parameters constrained
Δρ_max_ = 0.62 e Å^−3^
Δρ_min_ = −0.43 e Å^−3^

### 

Data collection: *SMART* (Bruker, 1997 ▶); cell refinement: *SAINT* (Bruker, 1997 ▶); data reduction: *SAINT*; program(s) used to solve structure: *SHELXS97* (Sheldrick, 2008 ▶); program(s) used to refine structure: *SHELXL97* (Sheldrick, 2008 ▶); molecular graphics: *SHELXTL* (Sheldrick, 2008 ▶); software used to prepare material for publication: *SHELXTL*.

## Supplementary Material

Crystal structure: contains datablocks I, global. DOI: 10.1107/S1600536808041354/ci2736sup1.cif

Structure factors: contains datablocks I. DOI: 10.1107/S1600536808041354/ci2736Isup2.hkl

Additional supplementary materials:  crystallographic information; 3D view; checkCIF report

## Figures and Tables

**Table 1:** Selected bond lengths (Å)

Fe1—N1	1.871 (2)
Fe1—N4	1.889 (2)
Fe1—N3	2.016 (2)
Fe1—N2	2.032 (2)
Fe1—Cl1	2.3080 (8)

**Table 2:** Hydrogen-bond geometry (Å, °)

*D*—H⋯*A*	*D*—H	H⋯*A*	*D*⋯*A*	*D*—H⋯*A*
C3—H3*A*⋯Cl1^i^	0.93	2.80	3.595 (4)	144
C10—H10*A*⋯Cl1^ii^	0.93	2.71	3.617 (3)	165
C11—H11*A*⋯O1^iii^	0.93	2.46	3.290 (5)	149

Symmetry codes: (i) 
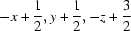
; (ii) 
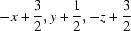
; (iii) 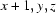.

**Fig. 1.**
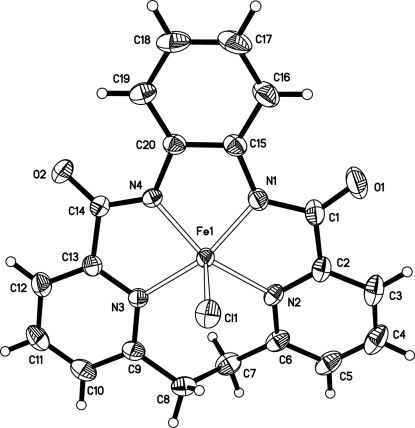
The molecular structure of the title compound. Displacement ellipsoids are drawn at the 30% probability level.

## supplementary crystallographic information

### Comment

The chemistry of macrocyclic complexes has attracted the interest of both inorganic and bioinorganic chemists in recent years. Iron(III) complexes are involved in numerous biological redox reactions performed by metalloenzymes (Momenteau *et al.*, 1994). As part of our studies on catalysis by N4 non-porphyrin complexes (Liu *et al.*, 2006; Yang *et al.*, 2007), we report here the crystal structure of a iron(III) complex with 1,2-[bis(6'-pyridine-2'-carboxamido)-ethane]benzene.

As shown in Fig.1, the complex has a five-coordinate structure with two pyridine and two deprotonated amide N atoms in the basal plane while the Cl ion is bonded to the Fe^III^ center in the apical position. The geometry around the Fe^III^ ion is approximately square-pyramidal. The Fe—N(amide) distances are shorter than the Fe—N(pyridine) distances (Table 1), both of which are shorter than the Fe—N distances found in the non-ring related Fe—N4 complexes such as [NEt_4_][Fe(bbpc)Cl_2_][H_2_bbpc is *N*,*N*'-(4,5-dichloro-*o*-phenylene)bis(4-tertbutylpyridine-2-carboxamide)] (Xu *et al.*, 2007). The Fe—Cl distance of 2.3080 (8) Å is slightly shorter than that observed in [Fe(bbpc)Cl_2_](Et_4_N) (2.3299 (9) Å and 2.3880 (9) Å), while it is longer than that in [FeCl(*meso*-NH_2_-octaethylporphyrin)] (2.2596 (8) Å, Sankar *et al.*, 2004).

In the crystalline state, the molecules are linked into a three-dimensional network by C—H···Cl and C—H···O hydrogen bonds (Table 2).

### Experimental

1,2-[Bis(6'-pyridine-2'carboxamido)-ethane]benzene (132 mg, 0.38 mmol) and sodium acetate (80 mg, 0.76 mmol) were added to a stirred solution of FeCl_3_.6H_2_O (244 mg, 0.9 mmol) in CH_3_OH (20 ml). The colour of the mixture turned green almost immediately. The mixture was refluxed for 3 h and dark green microcrystals appeared. They were collected by filtration, washed with methanol, and air-dried. (123 mg, yield 75%). Single crystals suitable for X-ray diffraction were grown *via* diffusion of Et_2_O into a DMF solution of the complex. Selected IR data (KBr, cm^-1^):*ν*=1629 (C=O), 1602 (C—N), 1572, 1346, 1287, 1142, 1083, 1081, 762. MS (FAB): 398.3([Fe(bpeb)]^+^).

### Refinement

All H atoms were positioned geometrically and refined as riding, with C-H = 0.93 Å (aromatic) or 0.97 Å (methylene) and *U*_iso_(H) = 1.2*U*_eq_(C).

### Crystal data

**Table:**

[Fe(C_20_H_14_N_4_O_2_)Cl]	*F*(000) = 884
*M**_r_* = 433.65	*D*_x_ = 1.600 Mg m^−^^3^
Monoclinic, *P*2_1_/*n*	Mo *K*α radiation, λ = 0.71073 Å
Hall symbol: -P 2yn	Cell parameters from 9989 reflections
*a* = 11.8532 (2) Å	θ = 2.2–27.4°
*b* = 8.2028 (1) Å	µ = 1.01 mm^−^^1^
*c* = 19.3507 (3) Å	*T* = 296 K
β = 106.889 (1)°	Plate, black
*V* = 1800.31 (5) Å^3^	0.44 × 0.16 × 0.10 mm
*Z* = 4	

### Data collection

**Table:**

Bruker SMART CCD area-detector diffractometer	4142 independent reflections
Radiation source: fine-focus sealed tube	3415 reflections with *I* > 2σ(*I*)
graphite	*R*_int_ = 0.041
φ and ω scans	θ_max_ = 27.5°, θ_min_ = 2.2°
Absorption correction: multi-scan (*SADABS*, Sheldrick, 1996)	*h* = −15→15
*T*_min_ = 0.778, *T*_max_ = 1.000	*k* = −10→10
24687 measured reflections	*l* = −25→25

### Refinement

**Table:**

Refinement on *F*^2^	Primary atom site location: structure-invariant direct methods
Least-squares matrix: full	Secondary atom site location: difference Fourier map
*R*[*F*^2^ > 2σ(*F*^2^)] = 0.040	Hydrogen site location: inferred from neighbouring sites
*wR*(*F*^2^) = 0.126	H-atom parameters constrained
*S* = 1.00	*w* = 1/[σ^2^(*F*_o_^2^) + (0.0642*P*)^2^ + 2.4383*P*] where *P* = (*F*_o_^2^ + 2*F*_c_^2^)/3
4142 reflections	(Δ/σ)_max_ = 0.001
253 parameters	Δρ_max_ = 0.62 e Å^−^^3^
0 restraints	Δρ_min_ = −0.43 e Å^−^^3^

### Special details

**Table:**

Geometry. All e.s.d.'s (except the e.s.d. in the dihedral angle between two l.s. planes) are estimated using the full covariance matrix. The cell e.s.d.'s are taken into account individually in the estimation of e.s.d.'s in distances, angles and torsion angles; correlations between e.s.d.'s in cell parameters are only used when they are defined by crystal symmetry. An approximate (isotropic) treatment of cell e.s.d.'s is used for estimating e.s.d.'s involving l.s. planes.
Refinement. Refinement of *F*^2^ against ALL reflections. The weighted *R*-factor *wR* and goodness of fit *S* are based on *F*^2^, conventional *R*-factors *R* are based on *F*, with *F* set to zero for negative *F*^2^. The threshold expression of *F*^2^ > σ(*F*^2^) is used only for calculating *R*-factors(gt) *etc*. and is not relevant to the choice of reflections for refinement. *R*-factors based on *F*^2^ are statistically about twice as large as those based on *F*, and *R*- factors based on ALL data will be even larger.

### Fractional atomic coordinates and isotropic or equivalent isotropic displacement parameters (Å^2^)

**Table:**

	*x*	*y*	*z*	*U*_iso_*/*U*_eq_	
Fe1	0.45008 (3)	0.56516 (5)	0.62759 (2)	0.02844 (13)	
Cl1	0.49959 (7)	0.48979 (10)	0.74759 (4)	0.0423 (2)	
O1	0.1136 (2)	0.6292 (4)	0.61432 (18)	0.0676 (8)	
O2	0.5587 (2)	0.2335 (3)	0.51016 (14)	0.0529 (6)	
N1	0.2870 (2)	0.5301 (3)	0.59827 (14)	0.0352 (5)	
N2	0.4003 (2)	0.7937 (3)	0.64688 (12)	0.0321 (5)	
N3	0.6194 (2)	0.5763 (3)	0.62630 (12)	0.0298 (5)	
N4	0.44925 (19)	0.3762 (3)	0.57162 (12)	0.0305 (5)	
C1	0.2181 (3)	0.6411 (4)	0.61888 (17)	0.0399 (7)	
C2	0.2867 (3)	0.7921 (4)	0.64770 (16)	0.0373 (6)	
C3	0.2337 (3)	0.9212 (4)	0.67153 (19)	0.0509 (9)	
H3A	0.1555	0.9156	0.6719	0.061*	
C4	0.3014 (4)	1.0606 (5)	0.6950 (2)	0.0564 (10)	
H4A	0.2696	1.1495	0.7127	0.068*	
C5	0.4145 (4)	1.0649 (4)	0.69173 (18)	0.0483 (8)	
H5A	0.4594	1.1585	0.7062	0.058*	
C6	0.4643 (3)	0.9306 (4)	0.66690 (16)	0.0377 (7)	
C7	0.5853 (3)	0.9393 (4)	0.65875 (18)	0.0443 (7)	
H7A	0.6159	1.0483	0.6716	0.053*	
H7B	0.5800	0.9222	0.6083	0.053*	
C8	0.6751 (3)	0.8146 (4)	0.70492 (17)	0.0418 (7)	
H8A	0.7468	0.8722	0.7301	0.050*	
H8B	0.6425	0.7683	0.7411	0.050*	
C9	0.7061 (2)	0.6787 (4)	0.66208 (15)	0.0352 (6)	
C10	0.8207 (3)	0.6559 (4)	0.65877 (18)	0.0453 (8)	
H10A	0.8794	0.7287	0.6824	0.054*	
C11	0.8485 (3)	0.5277 (5)	0.62112 (19)	0.0479 (8)	
H11A	0.9255	0.5124	0.6195	0.057*	
C12	0.7599 (3)	0.4217 (4)	0.58564 (17)	0.0413 (7)	
H12A	0.7760	0.3331	0.5600	0.050*	
C13	0.6471 (2)	0.4504 (3)	0.58911 (15)	0.0314 (6)	
C14	0.5471 (2)	0.3397 (4)	0.55255 (15)	0.0333 (6)	
C15	0.2484 (2)	0.3809 (4)	0.56301 (16)	0.0348 (6)	
C16	0.1338 (3)	0.3196 (4)	0.5414 (2)	0.0499 (8)	
H16A	0.0724	0.3773	0.5509	0.060*	
C17	0.1128 (3)	0.1721 (5)	0.5056 (2)	0.0590 (10)	
H17A	0.0364	0.1311	0.4905	0.071*	
C18	0.2030 (3)	0.0846 (4)	0.4919 (2)	0.0547 (9)	
H18A	0.1870	−0.0153	0.4683	0.066*	
C19	0.3178 (3)	0.1438 (4)	0.51280 (16)	0.0414 (7)	
H19A	0.3789	0.0840	0.5041	0.050*	
C20	0.3393 (2)	0.2947 (3)	0.54712 (15)	0.0328 (6)	

### Atomic displacement parameters (Å^2^)

**Table:**

	*U*^11^	*U*^22^	*U*^33^	*U*^12^	*U*^13^	*U*^23^
Fe1	0.0262 (2)	0.0225 (2)	0.0375 (2)	−0.00026 (14)	0.01073 (15)	−0.00375 (15)
Cl1	0.0483 (4)	0.0381 (4)	0.0427 (4)	0.0044 (3)	0.0169 (3)	0.0083 (3)
O1	0.0352 (13)	0.0650 (18)	0.109 (2)	0.0042 (12)	0.0307 (14)	−0.0117 (17)
O2	0.0472 (13)	0.0475 (14)	0.0671 (15)	0.0005 (11)	0.0215 (11)	−0.0268 (12)
N1	0.0278 (11)	0.0299 (13)	0.0484 (14)	0.0000 (9)	0.0119 (10)	−0.0014 (10)
N2	0.0410 (13)	0.0221 (11)	0.0345 (12)	0.0034 (10)	0.0132 (10)	−0.0013 (9)
N3	0.0287 (11)	0.0274 (12)	0.0334 (11)	−0.0026 (9)	0.0091 (9)	−0.0020 (9)
N4	0.0301 (11)	0.0237 (11)	0.0370 (12)	−0.0025 (9)	0.0088 (9)	−0.0049 (9)
C1	0.0337 (15)	0.0377 (17)	0.0515 (17)	0.0065 (12)	0.0175 (13)	0.0020 (14)
C2	0.0424 (16)	0.0333 (15)	0.0396 (15)	0.0099 (12)	0.0173 (12)	0.0021 (12)
C3	0.058 (2)	0.047 (2)	0.055 (2)	0.0213 (17)	0.0282 (17)	0.0042 (16)
C4	0.083 (3)	0.0406 (19)	0.0494 (19)	0.0225 (19)	0.0254 (18)	−0.0016 (15)
C5	0.076 (2)	0.0272 (15)	0.0401 (16)	0.0053 (15)	0.0136 (16)	−0.0027 (13)
C6	0.0547 (18)	0.0249 (14)	0.0325 (14)	0.0017 (13)	0.0108 (12)	0.0010 (11)
C7	0.0553 (19)	0.0264 (15)	0.0517 (18)	−0.0103 (13)	0.0166 (15)	−0.0024 (13)
C8	0.0421 (16)	0.0388 (17)	0.0411 (16)	−0.0095 (13)	0.0069 (13)	−0.0082 (13)
C9	0.0322 (14)	0.0346 (15)	0.0365 (14)	−0.0063 (12)	0.0066 (11)	−0.0002 (12)
C10	0.0314 (15)	0.051 (2)	0.0500 (18)	−0.0110 (14)	0.0058 (13)	0.0020 (15)
C11	0.0270 (14)	0.063 (2)	0.0540 (19)	0.0007 (14)	0.0126 (13)	0.0059 (17)
C12	0.0330 (15)	0.0472 (19)	0.0462 (17)	0.0054 (13)	0.0154 (13)	0.0001 (14)
C13	0.0311 (13)	0.0311 (14)	0.0321 (13)	0.0019 (11)	0.0092 (10)	0.0020 (11)
C14	0.0339 (14)	0.0296 (14)	0.0360 (14)	0.0024 (11)	0.0094 (11)	−0.0030 (11)
C15	0.0318 (14)	0.0287 (14)	0.0423 (15)	−0.0045 (11)	0.0084 (11)	0.0036 (12)
C16	0.0325 (16)	0.0450 (19)	0.070 (2)	−0.0058 (14)	0.0115 (15)	0.0041 (17)
C17	0.0431 (19)	0.047 (2)	0.079 (3)	−0.0221 (16)	0.0051 (17)	0.0039 (19)
C18	0.063 (2)	0.0346 (18)	0.059 (2)	−0.0211 (16)	0.0073 (17)	−0.0051 (15)
C19	0.0493 (18)	0.0317 (16)	0.0420 (16)	−0.0082 (13)	0.0114 (13)	−0.0036 (13)
C20	0.0326 (14)	0.0281 (14)	0.0360 (14)	−0.0047 (11)	0.0073 (11)	0.0012 (11)

### Geometric parameters (Å, °)

**Table:**

Fe1—N1	1.871 (2)	C7—C8	1.557 (5)
Fe1—N4	1.889 (2)	C7—H7A	0.97
Fe1—N3	2.016 (2)	C7—H7B	0.97
Fe1—N2	2.032 (2)	C8—C9	1.497 (4)
Fe1—Cl1	2.3080 (8)	C8—H8A	0.97
O1—C1	1.220 (4)	C8—H8B	0.97
O2—C14	1.231 (3)	C9—C10	1.392 (4)
N1—C1	1.358 (4)	C10—C11	1.373 (5)
N1—C15	1.412 (4)	C10—H10A	0.93
N2—C6	1.348 (4)	C11—C12	1.383 (5)
N2—C2	1.350 (4)	C11—H11A	0.93
N3—C9	1.352 (4)	C12—C13	1.378 (4)
N3—C13	1.353 (4)	C12—H12A	0.93
N4—C14	1.349 (4)	C13—C14	1.498 (4)
N4—C20	1.418 (3)	C15—C16	1.394 (4)
C1—C2	1.498 (5)	C15—C20	1.396 (4)
C2—C3	1.378 (4)	C16—C17	1.380 (5)
C3—C4	1.395 (6)	C16—H16A	0.93
C3—H3A	0.93	C17—C18	1.376 (6)
C4—C5	1.361 (6)	C17—H17A	0.93
C4—H4A	0.93	C18—C19	1.390 (5)
C5—C6	1.399 (4)	C18—H18A	0.93
C5—H5A	0.93	C19—C20	1.392 (4)
C6—C7	1.490 (5)	C19—H19A	0.93
			
N1—Fe1—N4	82.38 (10)	C8—C7—H7B	108.5
N1—Fe1—N3	161.28 (10)	H7A—C7—H7B	107.5
N4—Fe1—N3	82.51 (9)	C9—C8—C7	114.1 (3)
N1—Fe1—N2	82.39 (10)	C9—C8—H8A	108.7
N4—Fe1—N2	155.32 (10)	C7—C8—H8A	108.7
N3—Fe1—N2	107.69 (10)	C9—C8—H8B	108.7
N1—Fe1—Cl1	101.67 (8)	C7—C8—H8B	108.7
N4—Fe1—Cl1	108.32 (8)	H8A—C8—H8B	107.6
N3—Fe1—Cl1	93.53 (7)	N3—C9—C10	120.1 (3)
N2—Fe1—Cl1	93.71 (7)	N3—C9—C8	118.3 (3)
C1—N1—C15	125.8 (3)	C10—C9—C8	121.7 (3)
C1—N1—Fe1	117.7 (2)	C11—C10—C9	121.0 (3)
C15—N1—Fe1	116.19 (19)	C11—C10—H10A	119.5
C6—N2—C2	119.0 (3)	C9—C10—H10A	119.5
C6—N2—Fe1	130.7 (2)	C10—C11—C12	118.7 (3)
C2—N2—Fe1	109.69 (19)	C10—C11—H11A	120.6
C9—N3—C13	118.7 (2)	C12—C11—H11A	120.6
C9—N3—Fe1	129.3 (2)	C13—C12—C11	118.4 (3)
C13—N3—Fe1	111.65 (18)	C13—C12—H12A	120.8
C14—N4—C20	125.7 (2)	C11—C12—H12A	120.8
C14—N4—Fe1	118.49 (18)	N3—C13—C12	123.1 (3)
C20—N4—Fe1	115.45 (18)	N3—C13—C14	115.6 (2)
O1—C1—N1	127.6 (3)	C12—C13—C14	121.3 (3)
O1—C1—C2	121.5 (3)	O2—C14—N4	127.6 (3)
N1—C1—C2	110.8 (2)	O2—C14—C13	121.2 (3)
N2—C2—C3	123.4 (3)	N4—C14—C13	111.2 (2)
N2—C2—C1	116.0 (2)	C16—C15—C20	119.9 (3)
C3—C2—C1	120.6 (3)	C16—C15—N1	127.5 (3)
C2—C3—C4	117.6 (3)	C20—C15—N1	112.5 (2)
C2—C3—H3A	121.2	C17—C16—C15	119.0 (3)
C4—C3—H3A	121.2	C17—C16—H16A	120.5
C5—C4—C3	119.1 (3)	C15—C16—H16A	120.5
C5—C4—H4A	120.4	C18—C17—C16	121.1 (3)
C3—C4—H4A	120.4	C18—C17—H17A	119.4
C4—C5—C6	121.0 (3)	C16—C17—H17A	119.4
C4—C5—H5A	119.5	C17—C18—C19	120.8 (3)
C6—C5—H5A	119.5	C17—C18—H18A	119.6
N2—C6—C5	119.8 (3)	C19—C18—H18A	119.6
N2—C6—C7	119.2 (3)	C18—C19—C20	118.5 (3)
C5—C6—C7	120.9 (3)	C18—C19—H19A	120.7
C6—C7—C8	115.3 (3)	C20—C19—H19A	120.7
C6—C7—H7A	108.5	C19—C20—C15	120.6 (3)
C8—C7—H7A	108.5	C19—C20—N4	127.0 (3)
C6—C7—H7B	108.5	C15—C20—N4	112.4 (2)
			
N4—Fe1—N1—C1	−176.9 (2)	C2—N2—C6—C7	−173.6 (3)
N3—Fe1—N1—C1	−140.5 (3)	Fe1—N2—C6—C7	16.6 (4)
N2—Fe1—N1—C1	−16.3 (2)	C4—C5—C6—N2	−1.0 (5)
Cl1—Fe1—N1—C1	75.9 (2)	C4—C5—C6—C7	175.9 (3)
N4—Fe1—N1—C15	9.6 (2)	N2—C6—C7—C8	−63.1 (4)
N3—Fe1—N1—C15	46.0 (4)	C5—C6—C7—C8	119.9 (3)
N2—Fe1—N1—C15	170.1 (2)	C6—C7—C8—C9	107.4 (3)
Cl1—Fe1—N1—C15	−97.6 (2)	C13—N3—C9—C10	1.6 (4)
N1—Fe1—N2—C6	−173.7 (3)	Fe1—N3—C9—C10	174.0 (2)
N4—Fe1—N2—C6	−121.4 (3)	C13—N3—C9—C8	−178.2 (3)
N3—Fe1—N2—C6	−9.9 (3)	Fe1—N3—C9—C8	−5.7 (4)
Cl1—Fe1—N2—C6	85.0 (2)	C7—C8—C9—N3	−62.5 (4)
N1—Fe1—N2—C2	15.76 (19)	C7—C8—C9—C10	117.7 (3)
N4—Fe1—N2—C2	68.1 (3)	N3—C9—C10—C11	−1.8 (5)
N3—Fe1—N2—C2	179.57 (18)	C8—C9—C10—C11	177.9 (3)
Cl1—Fe1—N2—C2	−85.53 (19)	C9—C10—C11—C12	0.7 (5)
N1—Fe1—N3—C9	150.2 (3)	C10—C11—C12—C13	0.6 (5)
N4—Fe1—N3—C9	−173.5 (3)	C9—N3—C13—C12	−0.3 (4)
N2—Fe1—N3—C9	29.6 (3)	Fe1—N3—C13—C12	−174.0 (2)
Cl1—Fe1—N3—C9	−65.4 (2)	C9—N3—C13—C14	178.4 (2)
N1—Fe1—N3—C13	−37.0 (4)	Fe1—N3—C13—C14	4.7 (3)
N4—Fe1—N3—C13	−0.60 (19)	C11—C12—C13—N3	−0.8 (5)
N2—Fe1—N3—C13	−157.53 (18)	C11—C12—C13—C14	−179.4 (3)
Cl1—Fe1—N3—C13	107.44 (18)	C20—N4—C14—O2	1.6 (5)
N1—Fe1—N4—C14	164.8 (2)	Fe1—N4—C14—O2	−171.3 (3)
N3—Fe1—N4—C14	−4.1 (2)	C20—N4—C14—C13	−179.7 (2)
N2—Fe1—N4—C14	112.5 (3)	Fe1—N4—C14—C13	7.5 (3)
Cl1—Fe1—N4—C14	−95.4 (2)	N3—C13—C14—O2	171.0 (3)
N1—Fe1—N4—C20	−8.8 (2)	C12—C13—C14—O2	−10.3 (4)
N3—Fe1—N4—C20	−177.7 (2)	N3—C13—C14—N4	−7.8 (4)
N2—Fe1—N4—C20	−61.1 (3)	C12—C13—C14—N4	170.9 (3)
Cl1—Fe1—N4—C20	91.03 (19)	C1—N1—C15—C16	1.3 (5)
C15—N1—C1—O1	4.1 (6)	Fe1—N1—C15—C16	174.3 (3)
Fe1—N1—C1—O1	−168.8 (3)	C1—N1—C15—C20	178.5 (3)
C15—N1—C1—C2	−174.1 (3)	Fe1—N1—C15—C20	−8.6 (3)
Fe1—N1—C1—C2	13.1 (3)	C20—C15—C16—C17	1.3 (5)
C6—N2—C2—C3	−3.4 (4)	N1—C15—C16—C17	178.3 (3)
Fe1—N2—C2—C3	168.4 (3)	C15—C16—C17—C18	0.7 (6)
C6—N2—C2—C1	175.0 (3)	C16—C17—C18—C19	−1.0 (6)
Fe1—N2—C2—C1	−13.2 (3)	C17—C18—C19—C20	−0.8 (5)
O1—C1—C2—N2	−176.9 (3)	C18—C19—C20—C15	2.8 (5)
N1—C1—C2—N2	1.3 (4)	C18—C19—C20—N4	−179.2 (3)
O1—C1—C2—C3	1.5 (5)	C16—C15—C20—C19	−3.1 (5)
N1—C1—C2—C3	179.8 (3)	N1—C15—C20—C19	179.5 (3)
N2—C2—C3—C4	0.8 (5)	C16—C15—C20—N4	178.7 (3)
C1—C2—C3—C4	−177.5 (3)	N1—C15—C20—N4	1.3 (4)
C2—C3—C4—C5	1.6 (5)	C14—N4—C20—C19	15.3 (5)
C3—C4—C5—C6	−1.5 (5)	Fe1—N4—C20—C19	−171.7 (2)
C2—N2—C6—C5	3.4 (4)	C14—N4—C20—C15	−166.7 (3)
Fe1—N2—C6—C5	−166.4 (2)	Fe1—N4—C20—C15	6.4 (3)

### Hydrogen-bond geometry (Å, °)

**Table:**

*D*—H···*A*	*D*—H	H···*A*	*D*···*A*	*D*—H···*A*
C3—H3A···Cl1^i^	0.93	2.80	3.595 (4)	144
C10—H10A···Cl1^ii^	0.93	2.71	3.617 (3)	165
C11—H11A···O1^iii^	0.93	2.46	3.290 (5)	149

Symmetry codes: (i) −*x*+1/2, *y*+1/2, −*z*+3/2; (ii) −*x*+3/2, *y*+1/2, −*z*+3/2; (iii) *x*+1, *y*, *z*.
